# Genome-wide DNA methylation analysis of the porcine hypothalamus-pituitary-ovary axis

**DOI:** 10.1038/s41598-017-04603-x

**Published:** 2017-06-27

**Authors:** Xiao-Long Yuan, Zhe Zhang, Bin Li, Ning Gao, Hao Zhang, Per Torp Sangild, Jia-Qi Li

**Affiliations:** 1 Guangdong Provincial Key Lab of Agro-Animal Genomics and Molecular Breeding, National Engineering Research Centre for Breeding Swine Industry, College of Animal Science, South China Agricultural University, Guangzhou, Guangdong China; 2 Section of Comparative Pediatrics and Nutrition, Department of Veterinary Clinical and Animal Sciences, University of Copenhagen, Frederiksberg, Denmark; 3 Department of Animal Sciences, Georg-August University, Albrecht Thaer-Weg 3, Göttingen, Germany

## Abstract

Previous studies have suggested that DNA methylation in both CpG and CpH (where H = C, T or A) contexts plays a critical role in biological functions of different tissues. However, the genome-wide DNA methylation patterns of porcine hypothalamus-pituitary-ovary (HPO) tissues remain virtually unexplored. In this study, methylomes of HPO tissues were profiled to investigate their differences and similarities. We found that HPO methylomes displayed tissue-specific methylation patterns in both CpG and CpH contexts. At gene locations, the methylation and density of CpGs was negatively linked at transcription start sites but positively linked at transcription end sites. The densities of CpGs and CpHs at CpG island (CGI) locations were negatively correlated with their methylation. Moreover, the methylation interactions between CGIs and genes showed similar pattern in the CpG context but tissue-specific pattern in the CpH context. CpGs located in CGIs, upstream regions and exons were protected from methylation dynamics, whereas CGI shores, CGI shelves and intergenic regions were more likely to be targets of methylation changes. The methylation dynamics enriching in a tissue-specific manner appeared to maintain and establish the biological functions of HPO tissues. Our analyses provided valuable insights into the tissue-specific methylomes of porcine HPO tissues.

## Introduction

DNA methylation is one of the best understood epigenetic regulatory mechanisms^1^. There has recently been rapid development of biotechnology for the detection of DNA methylation, and genome-wide DNA methylation pattern profiles have been described for a number of species^2–4^, tissues^5^ and cell types^6^, even at a single-cell resolution^7^. These profiles have provided useful insights into the epigenetics of chromatin and the mechanisms of aging^8^, cell differentiation^9^, fetal development^5^ and tissue-specific maintenance^10^. In mammalian genomes, DNA methylation occurs predominantly at CpG sites. In general, CpGs within CpG islands (CGIs) and promoters, which are regions with a relatively high CpG content, are more likely to show hypomethylation^11^, whereas CpGs in gene bodies and outside CGIs show hypermethylation^12^. Both hypo- and hypermethylation are closely associated with transcription activities^13^. Furthermore, dynamic CpG methylation changes among different tissues are underrepresented in promoter and CGI regions, but overrepresented in gene bodies and outside CGIs^5, 14^, which supports the maintenance of tissue-specific functions.

The majority of non-CpG (CpH, where H = C, T or A) sites are unmethylated in mammalian genomes. However, several studies have revealed that CpH sites indeed become methylated and that their methylation is dynamic during the processes of brain development^14, 15^ and stem cell differentiation^16^. In the neuronal genome, during the development from a fetus to a young adult, CpH methylation increases to become the dominant form of methylation and is involved in the chromosome inactivation^15^. Moreover, CpH methylation has been reported to be negatively correlated with gene activity in the brain genomes of humans^15^, mice^17^ and birds (great tits)^18, 19^. These findings suggest that both CpG and CpH methylation play an important role during the mammalian development and morphogenesis.

According to a previous study^20^, CGIs are defined as the C-rich and G-rich genomic regions >= 200 bp in length, with a C + G content >= 50% and an observed/expected ratio of CpGs >= 0.6. In human and mouse genomes, CGIs encompass approximately 60% of gene promoters^20, 21^, and their conserved methylation levels and locations demonstrate that CGIs are the cis-regulatory sequences and serve as genome landmarks in evolution^20, 21^. Generally, CGIs that are located at gene promoters are protected from methylation dynamics and exhibit consistently low hypomethylation, which is closely associated with the active transcription^11, 22^. However, CGIs within gene bodies are frequently susceptible to methylation dynamics and display tissue-specific hypermethylation^21, 23^, which is associated with higher gene expression in somatic tissues^24, 25^, with the exception of the brain^18, 26^. Additionally, this hypermethylation of CGIs overlaps significantly with the trimethylation of H3K4 to regulate tissue-specific alternative transcripts^21, 23^. These observations suggest that the methylation of CGIs might interact closely with the biological functions of genes to determine tissue-specific methylation patterns.

In many regards, pigs and humans share similar genome and physiological characteristics, and previous studies have demonstrated that porcine genome-wide DNA methylation patterns are similar to those observed in humans. This finding supports the notion that pigs are a useful and stable biomedical model^12, 27^. In both pigs and humans, the hypothalamus-pituitary-ovary (HPO) axis is one of the key endocrine systems involved in the regulation of reproduction. The stimulation and regulation of gonadotropin-releasing hormone, gonadotropin and steroid hormone syntheses within the HPO axis are critical for the development and establishment of secondary sexual characteristics and the reproductive capacity. Nevertheless, few investigations have focused on the genome-wide DNA methylation profile of the porcine HPO axis.

To investigate the genome-scale DNA methylation of porcine HPO tissues, we profiled the methylomes of porcine HPO tissues using reduced representation bisulfite sequencing (RRBS), and compared their methylation profiles to describe the differences and similarities across HPO tissues in both CpG and CpH contexts. Next, we attempted to localize CGIs to gene locations and to characterize CGIs based on genic features to examine the interactions between genes and CGIs among HPO methylomes. Then the methylation dynamics of HPO tissues were explored, and these methylation dynamics were then associated with the biological functions of HPO tissues. These analyses provide valuable insights into the tissue-specific methylation pattern of porcine HPO tissues.

## Results

### Genome-wide DNA methylation of porcine HPO tissues in the CpG context

**Figure 1:**
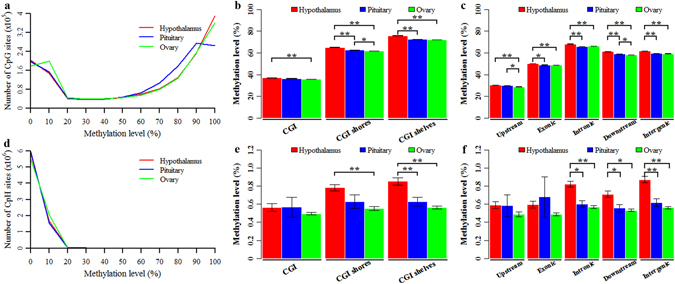
Genome-wide DNA methylation of porcine hypothalamus-pituitary-ovary tissues. Distribution of methylation levels in the CpG (**a**) and CpH (**d**) contexts. Mean methylation levels of CpGs in CGI-related regions (**b**) and gene-related regions (**c**). Mean methylation levels of CpHs in CGI-related regions (**e**) and gene-related regions (**f**). *Denotes p-value < 0.05, **denotes p-value < 0.01.

The detected CpG and CpH sites that were covered by at least five reads and coexisted across all tissues were considered for further analysis. The average methylation levels in the CpG context were 57.62%, 55.60% and 55.44% for the hypothalamus (H), pituitary (P) and ovary (O), respectively (see Supplementary Fig. S1a and Table S1). The CpG methylation levels in HPO tissues all presented a bimodal distribution (Fig. 1a), but the distributed features of these three tissues could be clearly distinguished from each other in the bimodal peaks. Comparison of the three tissues revealed that O exhibited the most CpGs with methylation levels ranging from 0 to 20% (O: 30.05%, P: 27.98%, H: 27.86%); P exhibited the most CpGs with methylation levels ranging from 60% to 90% (O: 31.38%, P: 39.93%, H: 31.76%); H exhibited the most CpGs with methylation levels higher than 90% (O: 25.82%, P: 18.84%, H: 27.78%) (Fig. 1a). The average methylation levels of the detected CpGs among the different genomic features are shown in Fig. 1b,c and Table S1. The average methylation levels of the different genomic features in H were all significantly higher than those in O (Fig. 1b,c). With the exception of CGIs and upstream regions, the average methylation levels of the genomic features in H were also significantly higher than those in P (Fig. 1b,c). The average methylation levels of CpGs located at CGIs and upstream regions displayed the lowest methylation levels, when compared with the other CGI- or gene-related regions (Fig. 1b,c, and Table S1), respectively.

### Genome-wide DNA methylation of porcine HPO tissues in the CpH context

Among HPO tissues, the distribution of CpH methylation levels was nearly the same, presenting a single-peak distribution, and more than 99.67% of the detected CpHs were hypomethylated (<20%) (Fig. 1d). The average CpH methylation level of H was 0.79%, which was higher than those of P (0.61%) and O (0.55%) (see Supplementary Fig. S1b and Table S1). The average methylation levels of the detected CpHs among different genomic features are shown in Fig. 1e,f and Table S1. The average methylation levels of CpHs located at CGIs and upstream regions in P were almost the same as in H (Fig. 1e,f, and Table S1), but higher than in O. The average methylation levels of CpHs located at CGIs and upstream regions were lower, when compared with those located in the other CGI- and gene-related regions (Fig. 1e,f and Table S1). Interestingly, the methylation level of CpHs located in exons in P was the highest when compared with those in H and O (Fig. 1e and Table S1). These results suggest that CpG and CpH methylation exhibits tissue-specific patterns among HPO methylomes.

### DNA methylation patterns of CpG and CpH in CGI and gene locations

**Figure 2:**
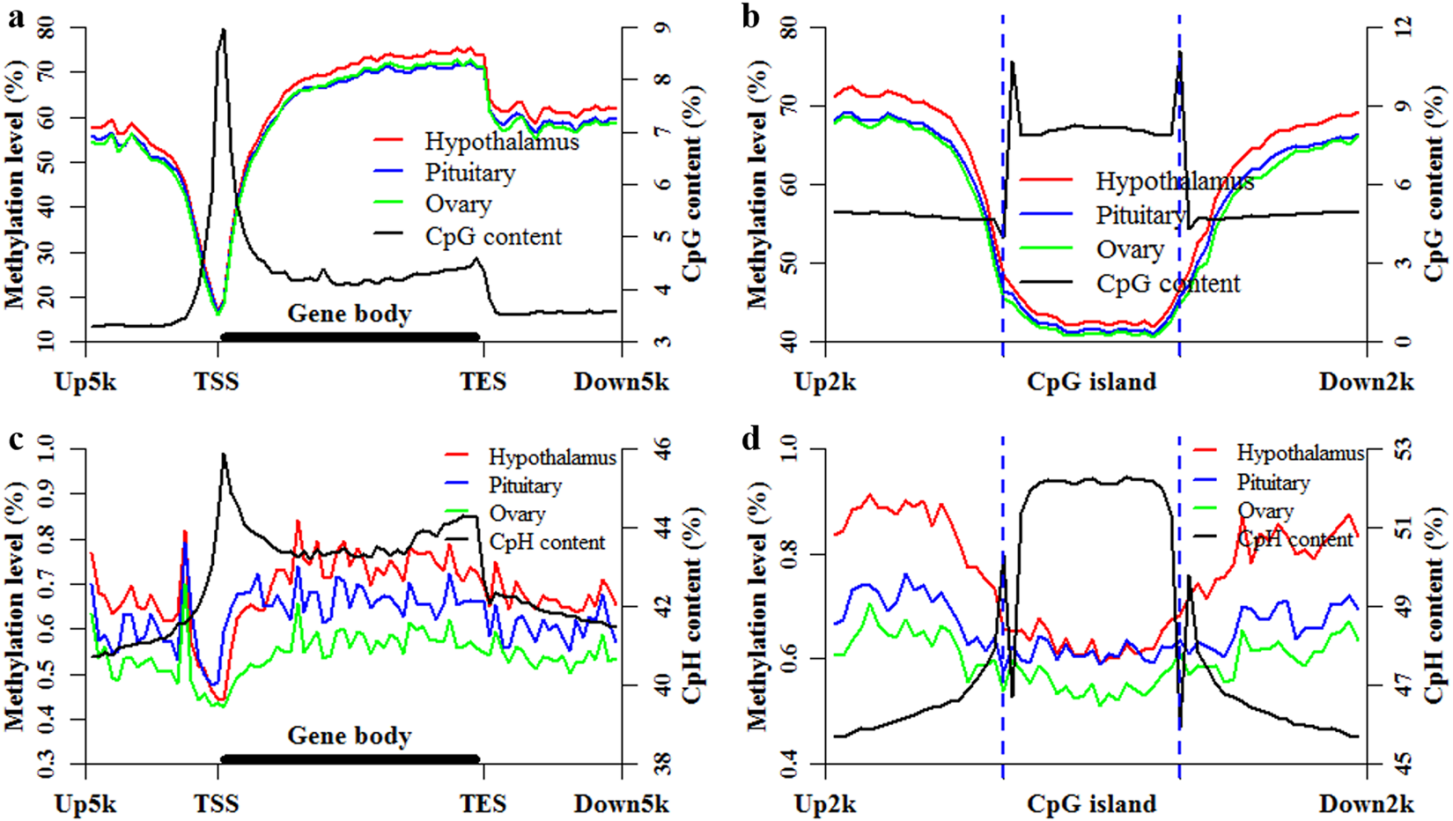
Methylation and density patterns of CpGs and CpHs at CGI and gene locations. Methylation and density patterns of CpG sites at the locations of genes (**a**) and CGIs (**b**). Methylation and density patterns of CpH sites at the locations of genes (**c**) and CGIs (**d**).

The methylation patterns of CpGs and CpHs were profiled to investigate the differences and similarities at the locations of genes and CGIs among HPO methylomes (Fig. 2). The DNA methylation levels at gene and CGI locations in H were higher, compared with that in P and O (Fig. 2a,b). Additionally, the DNA methylation levels at gene and CGI locations in P were almost the same as in O (Fig. 2a,b). In the CpH context, the methylation tendencies at gene and CGI locations in the HPO methylomes were similar to those for CpGs (Fig. 2c,d). H showed the highest CpH methylation level, and O displayed the lowest (Fig. 2c,d). Interestingly, the CpH methylation level in the beginning portion of the gene body was higher in P than in H (Fig. 2c). These results indicated that the methylation patterns of CpGs and CpHs were tissue specific.

### Correlation of DNA methylation with CpG and CpH densities

The densities of CpGs and CpHs, along with gene and CGI locations, were also determined to explore their correlation with DNA methylation (Fig. 2 and Table 1). We found that the CpG density was negatively correlated with the DNA methylation pattern at gene and CGI locations (Fig. 2a,b and Table 1). The CpG density was also negatively correlated with DNA methylation at the TSS (+/− 10 bins around TSS) (Fig. 2a and Table 1) but was positively correlated with DNA methylation at the TES (+/− 10 bins around TES) (Fig. 2a and Table 1). However, there was no obvious correlation between the density of CpHs and their methylation at gene locations, except in P (Fig. 2c and Table 1). Although the CpH density was not significantly associated with DNA methylation at the TSS (+/− 10 bins around TSS) (Fig. 2c and Table 1), it was significantly associated at the TES (+/− 10 bins around TES) (Fig. 2c and Table 1). Moreover, the density of CpHs within CGI locations were negatively correlated with their methylation patterns (Fig. 2d and Table 1).

**Table 1:** Correlation coefficients of DNA methylation with CpG and CpH densities.

Correlation coefficients	CpG methylation and density			CpH methylation and density		
	Hypothalamus	Pituitary	Ovary	Hypothalamus	Pituitary	Ovary
Gene locations	−0.47 (*P* = 9.41 × 10^−6^)	−0.47 (*P* = 1.16 × 10^−5^)	−0.44 (*P* = 4.34 × 10^−5^)	0.11 (*P* = 0.31)	0.39 (*P* = 3.32 × 10^−4^)	0.07 (*P* = 0.52)
TSS (+/− 10 bins around TSS)	−0.70 (*P* = 4.57 × 10^−4^)	−0.69 (*P* = 4.84 × 10^−4^)	−0.68 (*P* = 7.76 × 10^−4^)	−0.30 (*P* = 0.18)	0.16 (*P* = 0.49)	−0.30 (*P* = 0.12)
TES (+/− 10 bins around TES)	0.97 (*P* = 1.48 × 10^−13^)	0.98 (*P* = 3.63 × 10^−14^)	0.98 (*P* = 4.16 × 10^−14^)	0.78 (*P* = 4.88 × 10^−5^)	0.73 (*P* = 1.66 × 10^−4^)	0.64 (*P* = 1.62 × 10^−3^)
CGI locations	−0.80 (*P* = 1.27 × 10^−14^)	−0.80 (*P* = 2.84 × 10^−14^)	−0.79 (*P* = 1.05 × 10^−13^)	−0.90 (*P* = 4.95 × 10^−22^)	−0.72 (*P* = 5.82 × 10^−11^)	−0.80 (*P* = 1.93 × 10^−14^)

Correlation coefficients were calculated by Pearson’s correlation. Gene locations extended from Up5k to Down5k. CGI locations extended from Up2k to Down2k.

**Figure 3:**
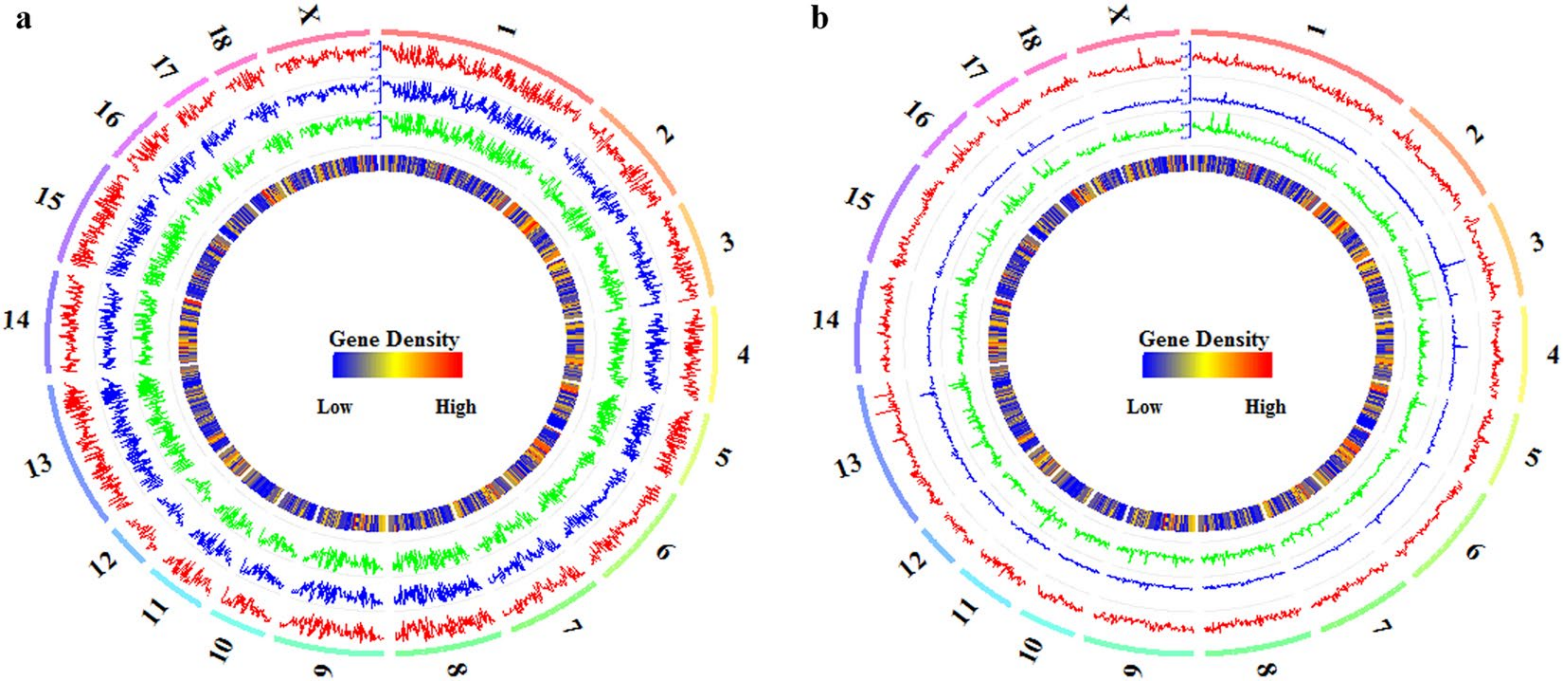
Global methylation of CpGs and CpHs and gene density. The global CpG (**a**) and CpH (**b**) methylation levels in the hypothalamus (track 1), pituitary (track 2) and ovary (track 3), from outside to inside, were quantified per 1 Mb window. The density of genes (track 4) was also quantified per 1 Mb window. The labels outside of track 1 represent the chromosomes of the porcine genome.

The correlations between the global CpG and CpH methylation of HPO tissues and the density of genes per 1 Mb window were explored (Fig. 3 and Supplementary Table S2). Among the porcine HPO methylomes, both CpG and CpH methylation appeared to be negatively associated with gene density (see Supplementary Table S2). Furthermore, in the CpG context, the Pearson’s correlation coefficients of H vs. P, H vs. O, and P vs. O were 0.92 (*P* < 2.22 × 10^−16^), 0.97 (*P* < 2.22 × 10^−16^) and 0.92 (*P* < 2.22 × 10^−16^), respectively. In the CpH context, the Pearson’s correlation coefficients were 0.40 (*P* < 2.22 × 10^−16^), 0.52 (*P* < 2.22 × 10^−16^) and 0.44 (*P* < 2.22 × 10^−16^) for H vs. P, H vs. O and P vs. O, respectively. The lower correlation coefficients in the CpH context compared with the CpG context indicate that the CpH methylomes are more variable than the CpG methylomes among HPO tissues.

### Methylation patterns of CGIs located at different genic features

**Figure 4:**
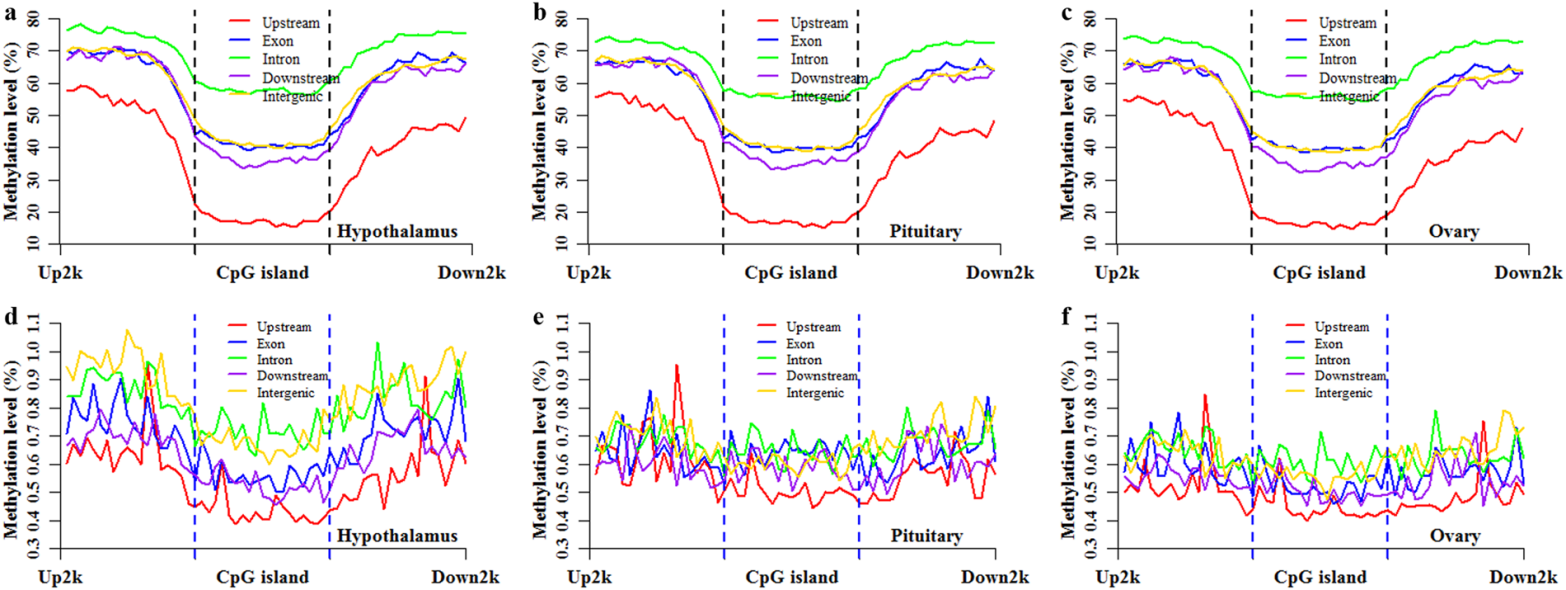
Methylation patterns of CGIs located in different genic features. The CpG methylation patterns of CGI locations in the hypothalamus (**a**), pituitary (**b**) and ovary (**c**). The CpH methylation patterns of CGI locations in the hypothalamus (**d**), pituitary (**e**) and ovary (**f**).

To investigate the interaction of the methylation between genes and CGIs among the porcine HPO methylomes, we divided the porcine genome into five genic features (upstream, exon, intron, downstream and intergenic) and then localized CGIs to these genic features (see Methods and Supplementary Fig. S2). The CpG and CpH methylation patterns of CGIs located at different genic features are depicted in Fig. 4, Supplementary Figs S3 and S4, to enable the evaluation of CpG and CpH methylation patterns of CGIs based on different genic features. In the CpG context, CGIs located at different genic features displayed different methylation patterns (Fig. 4a,b,c and Supplementary Fig. S3). The comparisons of the different genic features revealed that the methylation levels of CGIs located in upstream regions were the lowest (Fig. 4a), whereas those located in introns were the highest (Fig. 4a). Furthermore, the differences in methylation between CGIs and CGI shores were increased in upstream regions but reduced in introns (Fig. 4a and Supplementary Fig. S3). Compared with the whole CGIs (Fig. 2b), the methylation levels of CGIs located in exons and intergenic regions (Fig. 4a) exhibited small changes, but the methylation levels of CGIs located in downstream regions were decreased (Fig. 4a). These results suggested that genic features have an effect on the CpG methylation patterns of CGIs. Moreover, these effects displayed the same performance among the CpG methylomes of HPO axis (Fig. 4a,b and c).

The CpH methylation of CGIs based on different genic features also displayed different methylation patterns (Fig. 4d,e,f and Supplementary Fig. S4). Generally, comparisons of the different genic features revealed that the methylation levels of CGIs located in upstream regions were the lowest (Fig. 4d); the methylation levels of CGIs located in introns and intergenic regions were higher (Fig. 4d); the methylation levels of CGIs located in exons and downstream regions were intermediate (Fig. 4d). However, CGIs located in exons displayed higher methylation levels than those located in downstream and intergenic regions in P (Fig. 4e). The differences in methylation between CGIs and CGI shores were greater in H than in P and O (Fig. 4d,e and f). These results indicated that different genic features had different effects on the CpH methylation patterns of CGIs, and these effects showed different performances among the CpH methylomes of HPO axis (Fig. 4d,e and f).

### Methylation patterns of genes based on the different genic CGIs

**Figure 5:**
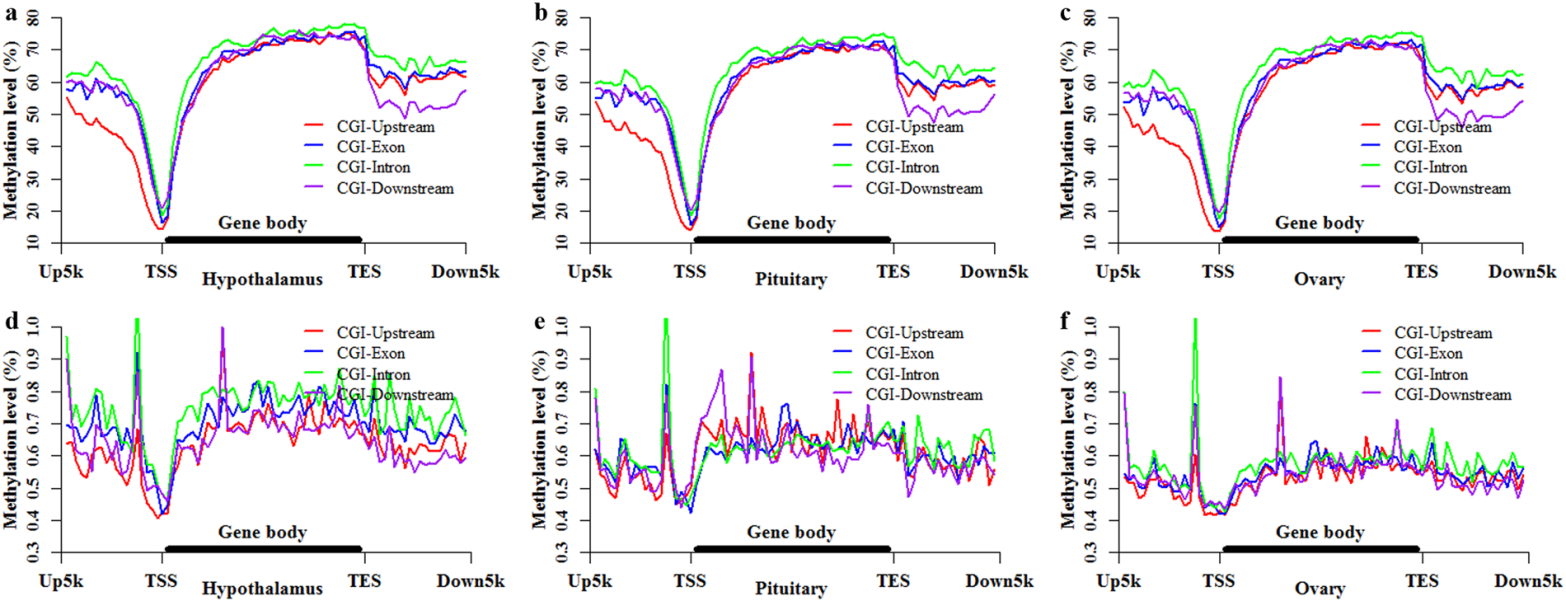
Methylation patterns of genes based on different genic CGIs. The CpG methylation patterns at gene locations based on the different genic CGIs in the hypothalamus (**a**), pituitary (**b**) and ovary (**c**). The CpH methylation patterns at gene locations based on the different genic CGIs in the hypothalamus (**d**), pituitary (**e**) and ovary (**f**). Genes were classified into CGI-Upstream, CGI-Exon, CGI-Intron and CGI-Downstream genes, basing on the localization of the CGIs.

The CpG and CpH methylation patterns of genes based on CGI locations are shown in Fig. 5, Supplementary Figs S5 and S6. The genes were classified into the CGI-Upstream, CGI-Exon, CGI-Intron and CGI-Downstream genes based on the localizations of CGIs (see Methods). In the CpG context, compared with the methylation pattern of whole genes (Fig. 2a), CGIs located at upstream regions tended to decrease the methylation level in upstream regions of the CGI-Upstream genes (Fig. 5a). CGIs located in exons had no clear influences on the methylation patterns of the CGI-Exon genes (Fig. 5a). CGIs located in introns appeared to increase the methylation level of the CGI-Intron genes (Fig. 5a). However, CGIs located in downstream regions appeared to decrease the methylation status in downstream regions of the CGI-Downstream genes. These results demonstrated that CGIs located in different genic features displayed different effects on the CpG methylation patterns of the associated genes. Moreover, these effects exhibited similar performance among CpG methylomes of HPO axis (Fig. 5a,b and c).

Compared with the CpH methylation patterns of whole genes (Fig. 2c), CGIs located in upstream and downstream regions appeared to reduce the CpH methylation levels of the CGI-Upstream and CGI-Downstream genes, and the CGIs located at introns tended to increase the CpH methylation levels of the CGI-Intron genes in the H methylome (Fig. 5d). However, in the P methylome, CGIs located in upstream and downstream regions appeared to increase the CpH methylation levels of the CGI-Upstream and CGI-Downstream genes, and the CGIs located in introns tended to reduce the CpH methylation levels of the CGI-Intron genes (Fig. 5e). Additionally, the CpH methylation levels at gene locations based on different CGI locations overlapped with each other in the O methylome (Fig. 5f). These results suggested that CGIs located in different genic features affected the CpH methylation patterns of associated genes, and these effects showed tissue-specific patterns among the CpH methylomes of HPO axis.

### Different CpG methylation patterns of porcine HPO methylomes

**Figure 6:**
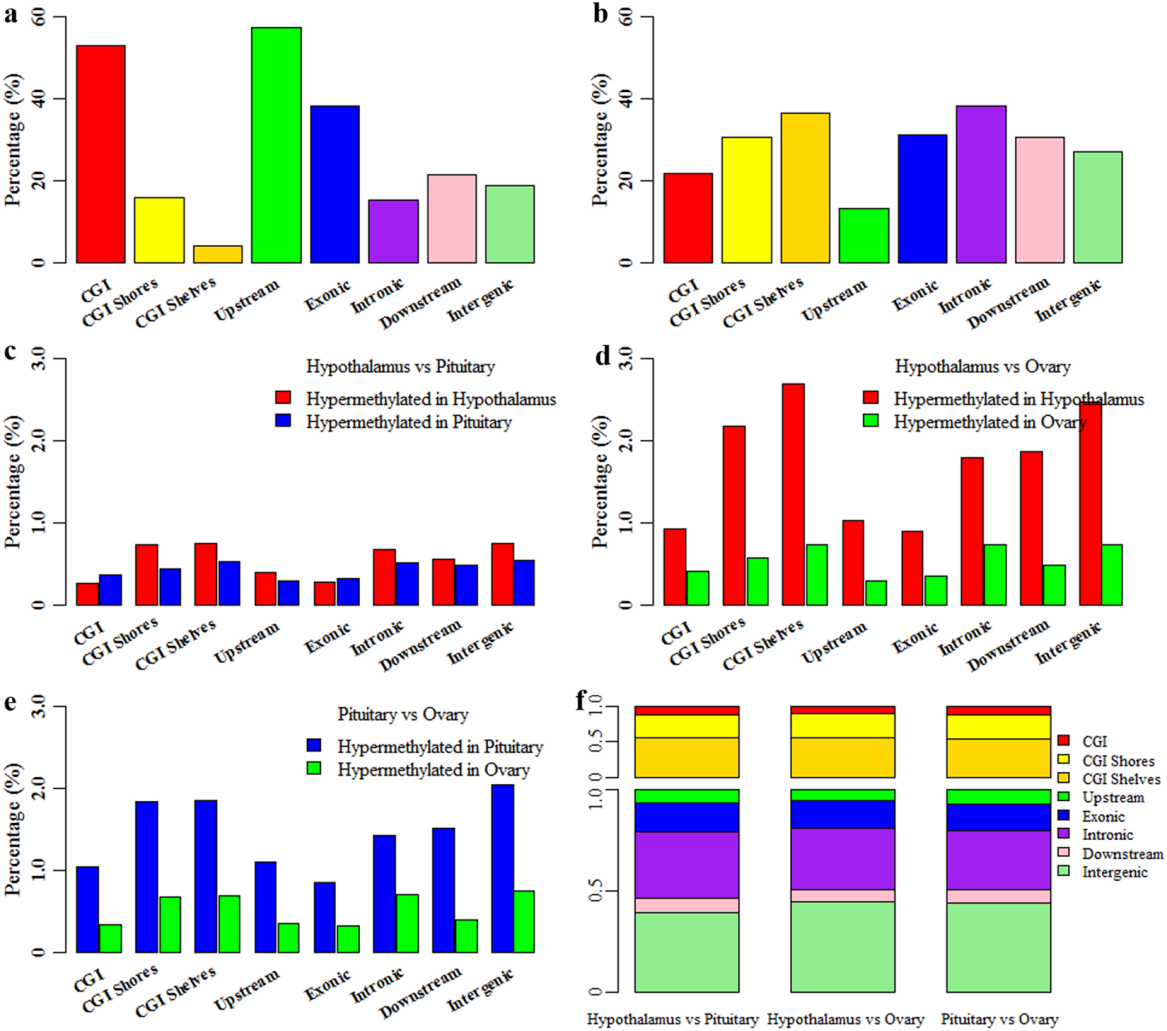
Differentially methylated CpGs and regions among HPO tissues. Distributions of the consistently hypomethylated CpG sites (**a**) and hypermethylated CpG sites (**b**) across HPO tissues. Distributions of differentially methylated CpG sites between the hypothalamus and pituitary (**c**), hypothalamus and ovary (**d**), and pituitary and ovary (**e**). Distributions of the differentially methylated regions among HPO tissues (**f**).

To explore the dynamic methylation of CpG sites among HPO tissues, we first counted the consistently hypomethylated CpG sites (HypoCs, methylation level <= 20%, Fig. 6a) and hypermethylated CpG sites (HyperCs, methylation level >= 80%, Fig. 6b) across the HPO methylomes. We found that HypoCs and HyperCs were distributed differently across CGI- and gene-related regions (Fig. 6a and b). Among the CpGs located at CGIs and upstream regions, 52.72% and 57.37% were the HypoCs, which was higher than for the CpGs located at other CGI- and gene-related regions (Fig. 6a). Moreover, 21.71% and 13.12% of the CpGs located at CGIs and upstream regions were the HyperCs, which was lower than for the CpGs located at other CGI- and gene-related regions (Fig. 6b).

Then, 14,744, 33,809 and 29,759 differentially methylated CpG sites (DMCs) were identified for H vs. P (Fig. 6c), H vs. O (Fig. 6d), and P vs. O (Fig. 6e), respectively, representing 1.05%, 2.41% and 2.12% of all detected CpGs (Table 2). In the comparisons of HPO methylomes, CGIs, upstream regions and exons showed a significant underrepresentation of DMCs (relative enrichment = 0.42–0.65, *P* < 2.22 × 10^−16^), but CGI shores, CGI shelves and intergenic regions showed a significant overrepresentation of DMCs (relative enrichment = 1.15–1.66, *P* ≤ 4.48 × 10^−12^) (Table 2). However, DMCs of introns and downstream regions enriched in a tissue-specific manner among the comparisons of H vs. P, H vs. O, and P vs. O (Table 2). These observations suggested that DMCs were more likely to appear at CGI shores, CGI shelves and intergenic regions, but were depleted at CGIs, upstream regions and exons. Furthermore, these DMCs were likely to be more hypermethylated in H and P than in O (Fig. 6d,e). We also found that there were more hypermethylated DMCs in H than in P, except at CGIs and exons (Fig. 6c).

**Table 2:** Distribution of differentially methylated CpGs among HPO methylomes.

	Detected CpGs	DMCs between Hypothalamus and Pituitary		DMCs between Hypothalamus and Ovary		DMCs between Pituitary and Ovary	
		Number	Enrichment	Number	Enrichment	Number	Enrichment
Total	1,403,700	14,744	—	33,809	—	29,759	—
CGI	556,039	3,509	0.48 (*P* < 2.22 × 10^−16^)	7,313	0.42 (*P* < 2.22 × 10^−16^)	7,612	0.52 (*P* < 2.22 × 10^−16^)
CGI shores	294,560	3,452	1.15 (*P* = 1.20 × 10^−12^)	8,044	1.18 (*P* < 2.22 × 10^−16^)	7,391	1.24 (*P* < 2.22 × 10^−16^)
CGI shelves	98,913	1,262	1.23 (*P* = 4.48 × 10^−12^)	3,383	1.47 (*P* < 2.22 × 10^−16^)	2,493	1.21 (*P* < 2.22 × 10^−16^)
Upstream	175,419	1,189	0.61 (*P* < 2.22 × 10^−16^)	2,308	0.51 (*P* < 2.22 × 10^−16^)	2,529	0.65 (*P* < 2.22 × 10^−16^)
Exon	228,324	1,340	0.51 (*P* < 2.22 × 10^−16^)	2,848	0.47 (*P* < 2.22 × 10^−16^)	2,661	0.51 (*P* < 2.22 × 10^−16^)
Intron	369,473	4,359	1.17 (*P* < 2.22 × 10^−16^)	9,303	1.06 (*P* = 9.39 × 10^−7^)	7,838	1.00 (*P* = 0.95)
Downstream	89,868	919	0.97 (*P* = 0.42)	2,114	0.98 (*P* = 0.27)	1,706	0.89 (*P* = 2.22 × 10^−6^)
Intergenic	540,616	6,937	1.42 (*P* < 2.2 × 10^−16^)	17,236	1.66 (*P* < 2.22 × 10^−16^)	15,025	1.63 (*P* < 2.22 × 10^−16^)

The enrichment of DMCs for certain genomic regions was using with a two-tail Fisher’s exact test.

### Biological functions of DMR genes in HPO tissues

Furthermore, 637, 1,884, and 1,511 differentially methylated regions (DMRs) were identified for H vs. P, H vs. O, and P vs. O, respectively (Fig. 6f). We found that DMRs were more likely to occur at CGI shores, CGI shelves, exons, introns and intergenic regions, but were likely to deplete at CGIs, upstream and downstream regions (Fig. 6f). To gain insight into the biological processes in which DMR genes might be involved, we performed the Gene Ontology (GO) enrichment analysis on genes whose upstream regions exhibited at least one DMR. The significant GO terms are shown in Supplementary Fig. S7. We found that the most significantly enriched terms were the morphogenesis (such as the organ morphogenesis, cell morphogenesis and anatomical structure morphogenesis), and development (such as the reproductive structure development, reproductive system development and vasculature development), cell differentiation and cellular developmental process. These results suggested that these DMR genes were enriched in a tissue-specific manner and were involved in biological functions of HPO tissues, indicating that DNA methylation might play an important role in establishing and maintaining the tissue-specific functions of HPO tissues.

## Discussion

The proper collaboration among HPO tissues is required for general developmental and reproductive processes in pigs. There are many reports demonstrating that DNA methylation plays an important role in the establishment and maintenance of tissue-specific functions^5, 28^ and morphogenesis^14, 29^. In this study, we profiled the genome-wide DNA methylation of porcine HPO tissues to describe and compare the similarities, differences and interactions of methylation between CGIs and genes among HPO methylomes. We found that the methylomes of HPO tissues are tissue-specific, and that the methylation patterns of CpGs and CpHs are highly associated with their densities at gene and CGI locations. CpH methylomes are more dynamic than CpG methylomes. CGIs located in different genic features display different methylation patterns. The interactions of the methylation of CGIs and genes showed similar patterns in the CpG context, but displayed tissue-specific patterns in the CpH context. The dynamics of CpG methylation are likely to deplete in CGIs, upstream regions and exons but occur more frequently in CGI shores, CGI shelves and intergenic regions (Table 2).

Among HPO methylomes, we found that methylation levels of CpGs and CpHs located at CGIs and upstream regions in H appeared to be equal to those in P but higher than that in O (Fig. 1b,c and Table S1). These specific methylation patterns of CGIs and upstream regions might be due to the different functions and morphologies of HPO tissues. We also found that the CpG located at CGIs and upstream regions were protected from methylation dynamics (Fig. 6 and Table 2). The HypoCs tended to be found in CGIs and upstream regions, rather than in other CGI- and gene-related regions (Fig. 6a), which was in line with results from 26 different human tissues^11^. Moreover, these HypoCs tended to be located near the TSSs and were highly associated with house-keeping genes^11^. Additionally, the HyperCs were depleted in CGIs and upstream regions, but occurred more frequently in other CGI- and gene-related regions (Fig. 6b). In total, 74.44% and 70.49% of the CpGs located at CGIs and upstream regions were HypoCs or HyperCs across the HPO methylomes; furthermore, the enrichments of DMCs located at CGIs and upstream regions ranged from 0.42 to 0.65 (Table 2), which were much lower than the enrichments of DMCs located CGI shores, CGI shelves and intergenic regions (ranging from 1.15 to 1.66, Table 2). These results suggest that the differences of the CpG methylation are overrepresented in CGIs and upstream regions but underrepresented in CGI shores, CGI shelves and intergenic regions. These observations indicate that CGIs and upstream regions are protected from methylation changes, which are consistent with previous studies of different human tissues^11, 22^.

Nevertheless, CpGs located at exons were also observed to be protected from methylation dynamics among HPO methylomes (Fig. 6 and Table 2). We found that 68.92% of CpGs located at exons were HypoCs or HyperCs (Fig. 6a,b). Among the comparisons of HPO methylomes, the enrichment of DMCs located at exons ranged from 0.47 to 0.51 (Table 2), which was much lower than the enrichments of DMCs located at CGI shores, CGI shelves, introns, downstream and intergenic regions. These results indicate that CpGs located at exons are also protected from the methylation dynamics among HPO methylomes, which was not consistent with previous observations made in human blood, brain, muscle and spleen tissues^30^, and 17 other human tissues^22^. Interestingly, there were more hypermethylated DMCs in exons in P than in H, but the numbers of the hypermethylated DMCs in introns, upstream, downstream and intergenic regions in P were lower than in H (Fig. 6c). Moreover, H3K36me3 modifications frequently occur at exons^31^, and the DNA methylation of exons is enriched with the binding of CTCF^32^ and MeCP2^33^, which affects alternative splicing and transcription. These observations suggest that the methylation of exons shows specific patterns among HPO methylomes and reveals a complex epigenetic role of exons in the maintenance and establishment of tissue-specific biological functions. In addition, among HPO tissues, we also found the CpG methylation level within introns was higher than in exons among HPO tissues (Fig. 1c). This result was in line with observations in other porcine tissues^12^ but contrasted with reports based on human genomes^34^. The contrasting methylation patterns of introns and exons observed in humans and pigs might be explained by the differences in the evolution of these two species. The differences in methylation between introns and exons also support their roles in regulating pre-mRNA splicing^33, 35^.

Among HPO methylomes, we found that CpH methylation (Fig. 3b) was more variable than CpG methylation (Fig. 3a). Moreover, interactions of the methylation of CGIs and genes were expressed in a tissue-specific manner among HPO methylomes in the CpH context (Figs 4 and 5). These observations highlight the potentially important epigenetic regulatory role of CpH methylation among HPO tissues. In this study, although the genome-wide DNA methylation patterns of HPO tissues were clearly illustrated, there were two main limitations. The first limitation was that the comparisons of different methylation profiles were only performed among HPO tissues. The tissue-specific DNA methylation patterns among porcine HPO tissues will become clearer upon comparison with other tissues. Another limitation was that although RRBS is suitable for accurately capturing a comprehensive and representative fraction of CpGs^36^ and CpHs^37^ throughout the genome, the characterization of tissue-specific DNA methylation patterns among HPO tissues would be more complete if we were able to improve the coverage of the whole genome.

The functions and morphologies of HPO tissues are different from each other, and these characteristics might result in different DNA methylation patterns among HPO tissues. In the CGI- and gene-related regions, the methylation levels of O appeared to be the lowest, whereas those of H appeared to be the highest in a cytosine context (Table S1). The CpG methylation levels of the HPO methylomes all exhibited a bimodal distribution (Fig. 1a), as observed in previous studies^38, 39^ using RRBS, but the distributed features of these three tissues were clearly different from each other in the bimodal peaks (Fig. 1a). In addition, the average methylation levels of the CGI and gene locations were distinct from each other (Fig. 2). These observations that different tissues show different methylation patterns are consistent with previous studies^30, 40^ and suggest that there might be tissue-specific epigenetic gene regulatory mechanisms among different tissues. In the CpG context, the methylation of CGIs located in upstream regions was lower, compared with those in exons, introns, downstream and intergenic regions (Fig. 4a). These results are in line with previous studies showing that promoter CGIs are hypomethylated^5^, whereas intergenic and intragenic CGIs are preferentially susceptible to methylation in different tissues of humans^21^ and mice^30^. In conclusion, among HPO methylomes, both CpGs and CpHs display tissue-specific methylation patterns, and these tissue-specific patterns might play a vital role in guiding the establishment and maintenance of tissue-specific characteristics and functions of HPO tissues.

## Methods

### Sample preparation and ethics statement

Three HPO tissues were collected from three female Landrace × Yorkshire crossed gilts aged 180 days. Pig cares and experiments were approved by the Animal care and Use Committer of South China Agricultural University, Guangzhou, China (approval number: SCAU#2013-10), and conductions were based on the Regulations for the Administration of Affairs Concerning Experimental Animals (Ministry of Science and Technology, China, revised in June 2004). Pigs were fed the same diet daily and reared in the same conditions and environments. HPO tissues were collected from these three pigs, and were frozen quickly in liquid nitrogen and then stored at −80 °C for further using.

### RRBS library and sequencing

The library constructions and sequencing services were provided by RiboBio Co., Ltd. (Guangzhou, China).The genomic DNA of these three HPO tissues were extracted using a DNeasy Blood & Tissue Kit (Qiagen, Beijing), and then, after checking on the quality of the extracted DNA, one library was built for each tissue based on previously published RRBS studies^36^. The processes and procedures of RRBS libraries were briefed as follows. Firstly, the purified genomic DNA was digested overnight with MspI (New England Biolabs, USA). For the MspI digested segments, the sticky ends were filled with CG nucleotides and 3′ A overhangs were added. Secondly, methylated Illumina sequencing adapters with 3′ T overhangs were ligated to the digested segments, and the products obtained were purified. Then 110–220 bp fragments were selected and converted by bisulfite using an EZ DNA Methylation Gold Kit (Zymo Research, USA). Lastly, libraries of 110–220 bp fragments were PCR amplified and each library was sequenced using one lane of an Illumina HiSeq 2500 and 100 bp paired-end reads. The first two nucleotides were trimmed from all the second read sequences to blunt-end the MspI site. All reads were trimmed using Trim Galore (v0.4.0) software (Babraham Bioinformatics, http://www.bioinformatics.babraham.ac.uk/projects/trim_galore/) and a Phred quality score of 20 as the minimum. The adaptor pollution reads and multiple N reads (where N >10% of one read) were removed to generate the clean reads. The quality control checks were performed by FastQC (v0.11.3) software (Babraham Bioinformatics). The clean reads were mapped to the porcine reference genome^41^ (Sscrofa 10.2, downloaded from Ensembl, http://www.ensembl.org/Sus_scrofa/Info/Index), and then call the DNA methylation by Bismark (v0.14.5)^42^ with default parameters. For the overlapped reads, only the methylation calls of read 1 were used for in the process by Bismark with the option “—no_overlap”, in order to avoid scoring the overlapping methylation calls twice. The bisulfite conversion rates were calculated as the number of covered CpHs, which were unconverted, was divided by the total number of covered CpHs^43^. The bisulfite conversion efficiencies of these nine libraries were 99.23%, 99.15%, 99.17%, 99.47%, 97.57%, 99.41%, 99.47%, 99.45%, 99.40% for hypothalamus 1, hypothalamus 2, hypothalamus 3, pituitary 1, pituitary 2, pituitary 3, ovary 1, ovary 2, ovary 3. Uniquely mapped reads were retained for further analyses. These nine RRBS data were submitted to European Nucleotide Archive (accession number: PRJEB16678). For cytosine sites, reads from specific strand where this cytosine located at were used to calculate the methylation levels.

### Annotation of CGI and gene location

Porcine CGI locations were downloaded from UCSC (http://hgdownload.soe.ucsc.edu/goldenPath/susScr3/database/). CGIs were described as regions >200 bp in length, with a C and G percentage >0.5, and a ratio of the observed CpG/expected CpG >0.6. The expected CpG was calculated as (GC content/2)^2^. The +/− 2 kb regions outside of CGIs were defined as CGI shores, and the +/− 2 kb regions outside of CGI shores were defined as CGI shelves. Gene locations were downloaded from Ensembl (http://www.ensembl.org/Sus_scrofa/Info/Index). Basing on gene locations of Ensembl, the porcine genome was separated into five genic features, which were upstream, exonic, intronic, downstream and intergenic regions (see Supplementary Fig. S2). The upstream region was 5 kb upstream region of the TSS. The exon was defined as the integration of 5′UTR, CDS and 3′UTR arranging from the TSS to the TES. The intron was determined as the integration of introns arranged from the TSS to the TES. The downstream region was 5 kb downstream region of the TES. The intergenic region was denoted as the outside regions of upstream, exonic, intronic and downstream regions.

According to previous studies on the localization of CGIs^44^ and fragments^45^ to genomic features, we localized CGIs to genic features. When more than 50% of a CGI overlapped with a specific genic feature, that CGI was classified with the specific genic feature (see Supplementary Fig. S2). For example, when the overlap ratio between a CGI and the upstream genome sequences was greater than 50%, that CGI was defined as an upstream CGI, and the related genes were referred to as the CGI-Upstream genes. When the overlap ratio between a CGI and exons was greater than 50%, that CGI was defined as an exonic CGI, and the related genes were referred to as the CGI-Exon genes.

### Analysis and calculation

After DNA methylation calling by Bismark for these nine RRBS data, 1,403,700 CpGs and 7,580,489 CpHs covered by at least five reads and coexisted across all tissues were remained for further analysis. The methylation level of a cytosine was calculated as the methylated reads of this cytosine divided the total covered reads. The methylation level of one kind of tissues was calculated by the average methylation level across the three replicates in each cytosine context. For the specific region, the methylation level was the average level of cytosines covered in this region. To profile the DNA methylation patterns at gene and CGI locations, the gene locations were divided into 20, 40 and 20 bins for Up5k, gene body and Down5k, respectively, and CGI locations were divided into 20, 20 and 20 bins for Up2k, CGIs and Down2k, respectively. These analyses were undertaken by Perl and R scripts.

The significant differences between two groups were tested using the Student’s test. Pearson’s correlation analysis was performed for all correlation analysis. The average CpG methylation and the numbers of genes were counted per 1 Mb window (on overlapping) to explore their Pearson’s correlation. The CpG methylation level of each 1 Mb window was calculated as the average methylation level of covered CpGs in this window. The gene density was the number of genes in each window. The DMCs and DMRs were calculated by the R package “DSS”^46, 47^. The CpGs, whose methylation levels changed more than 20%, were identified as DMCs according to a Wald test (*P* ≤ 0.01). DMRs were defined as the regions containing at least three continuing DMCs and more than 50 bp in length. The enrichment of DMCs for certain genomic regions was using with a two-tail Fisher’s exact test. The GO enrichment analysis of biological processes were undertaken by the R package “clusterProfiler”^48^ according to the over-representation test^49^ (*P* ≤ 0.05). The background of the GO enrichment analysis was the genes who exhibited at least one DMR.

## Electronic supplementary material

Supplementary Information
