# Supplementary material for: Genome-wide DNA methylation analysis of the porcine hypothalamus-pituitary-ovary axis
Source: Sci Rep. 2017 Jun 27;7:4277. doi: 10.1038/s41598-017-04603-x (PMC5487323; doi:10.1038/s41598-017-04603-x)
Supplement: Supplementary file 1 — Supplementary Information [file 41598_2017_4603_MOESM1_ESM.pdf]

Genome-wide DNA methylation analysis of the porcine hypothalamus-pituitary-ovary axis

Xiao-Long Yuan[1,2+], Zhe Zhang[1+], Bin Li[1], Ning Gao[1,3], Hao Zhang[1], Per Torp Sangild[2] and Jia-Qi Li[1*]

[1]Guangdong Provincial Key Lab of Agro-Animal Genomics and Molecular Breeding, National Engineering Research Centre for Breeding Swine Industry, College of Animal Science, South China Agricultural University, Guangzhou, Guangdong, China

[2]Section of Comparative Pediatrics and Nutrition, Department of Veterinary Clinical and Animal Sciences, University of Copenhagen, Frederiksberg, Denmark

[3]Department of Animal Sciences, Georg-August University, Albrecht Thaer-Weg 3, Göttingen, Germany

*Corresponding authors: Prof. Jia-Qi Li, E-mail: jqli@scau.edu.cn

[+]These authors contributed equally to this work.

Table S1 | Average methylation levels of different genomic features in HPO methylomes

	CpG methylation level			CpH methylation level		
	Hypothalamus	Pituitary	Ovary	Hypothalamus	Pituitary	Ovary
**Genome**	57.62%	55.60%	55.44%	0.79%	0.61%	0.55%
**Upstream**	30.45%	29.83%	28.74%	0.59%	0.58%	0.49%
**Exon**	50.06%	48.81%	48.74%	0.60%	0.68%	0.49%
**Intron**	68.22%	65.75%	66.16%	0.82%	0.60%	0.57%
**Downstream**	61.12%	58.96%	58.12%	0.71%	0.57%	0.53%
**Intergenic**	61.79%	59.33%	59.17%	0.87%	0.62%	0.56%
**CGI**	36.88%	36.35%	35.81%	0.56%	0.57%	0.49%
**CGI shores**	64.96%	62.43%	61.63%	0.78%	0.63%	0.55%
**CGI shelves**	75.68%	72.42%	72.11%	0.85%	0.63%	0.56%

Table S2 | Correlation coefficients between DNA methylation and gene density

Correlation Coefficients	CpG methylation			CpH methylation		
	Hypothalamus	Pituitary	Ovary	Hypothalamus	Pituitary	Ovary
**Gene density**	-0.079 ($P = 9.14\times10^{-5}$)	-0.073 ($P = 2.91\times10^{-4}$)	-0.079 ($P = 1.41\times10^{-7}$)	-0.19 ($P = 2.20\times10^{-16}$)	-0.03 ($P = 1.30\times10^{-1}$)	-0.11 ($P = 1.55\times10^{-8}$)

Correlation coefficients were calculated by Pearson's correlation. The DNA methylation and density of genes were quantified per 1 Mb window.

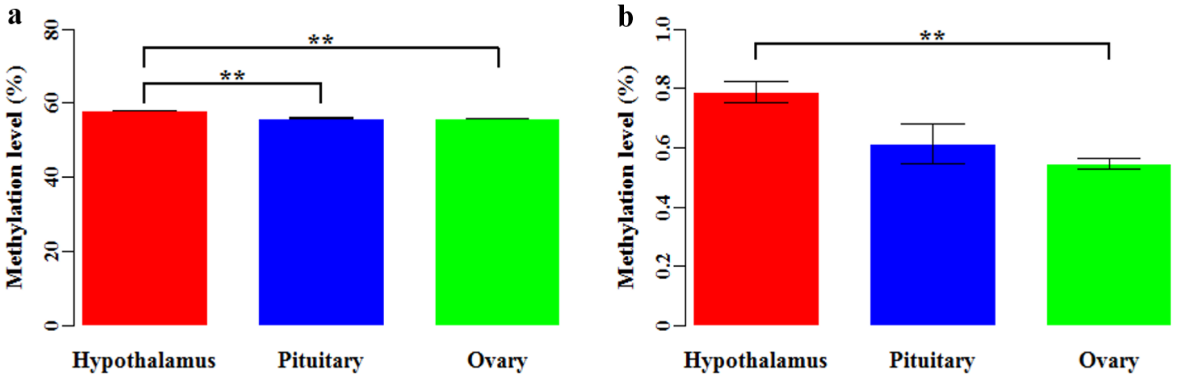

Figure S1 | Average methylation levels of hypothalamus-pituitary-ovary tissues in the CpG (a) and CpH (b) context. ** denotes p-value < 0.01

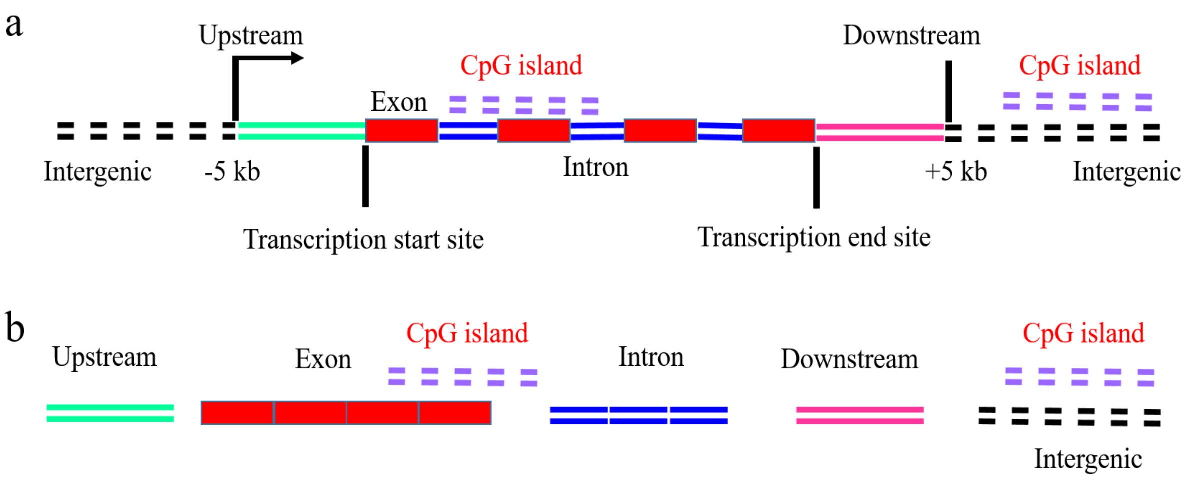

Figure S2 | Localizations of CGIs based on genic features. a) Gene locations were integrated and classified into upstream, exon, intron, downstream and intergenic regions. **b**) When more than 50% of a CGI overlapped with a specific genic feature, that CGI was classified with the specific genic feature. For example, when the overlap ratio between a CGI and the upstream genome sequences was greater than 50%, that CGI was defined as an upstream CGI, and the related genes were referred to as the CGI-Upstream genes. When the overlap ratio between a CGI and exons was greater than 50%, that CGI was defined as an exonic CGI, and the related genes were referred to as the CGI-Exon genes

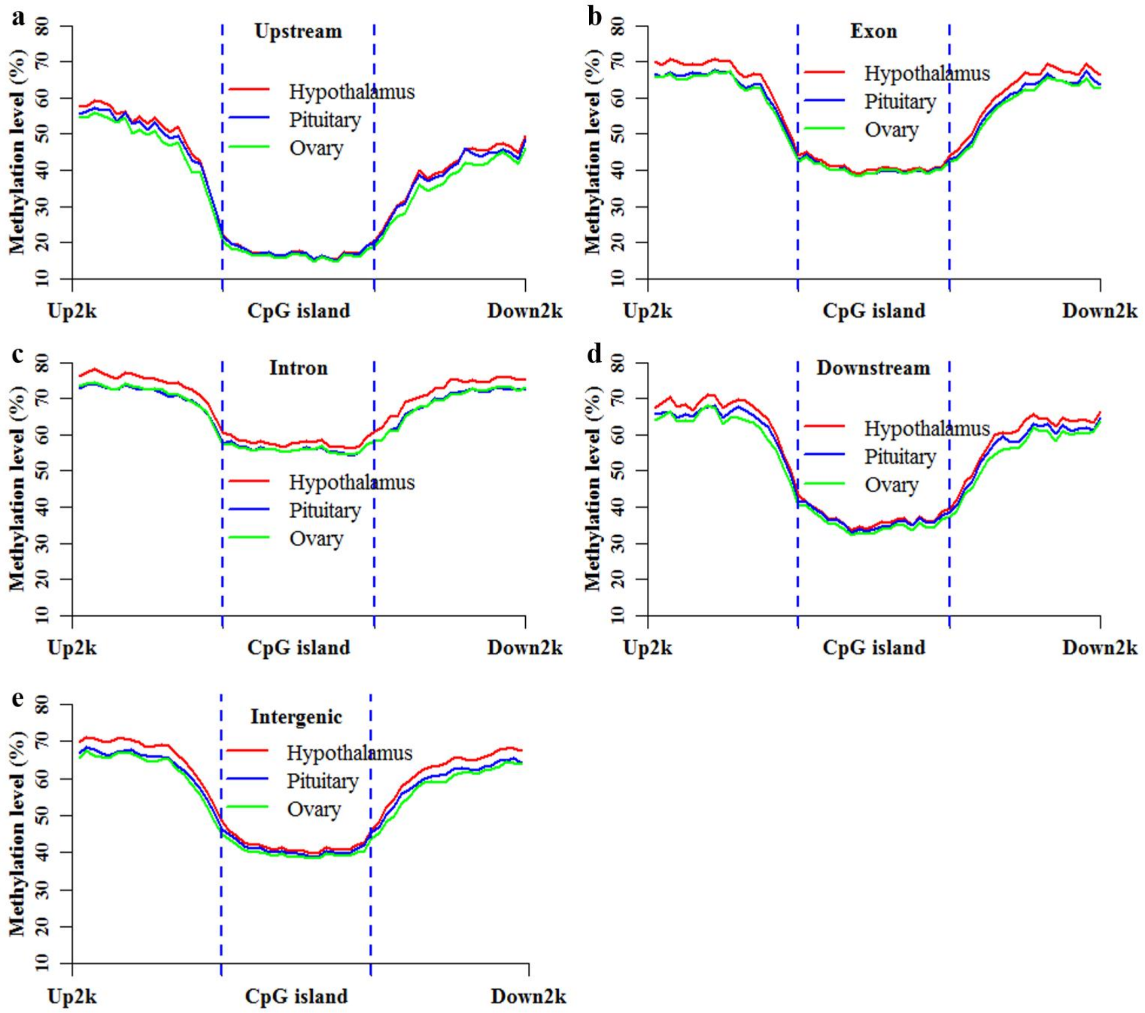

Figure S3 | Methylation patterns of CGIs located in different genic features in the CpG context. The methylation patterns of CGIs which located in upstream regions (**a**), exons (**b**), introns (**c**), downstream regions (**d**) and intergenic regions (**e**).

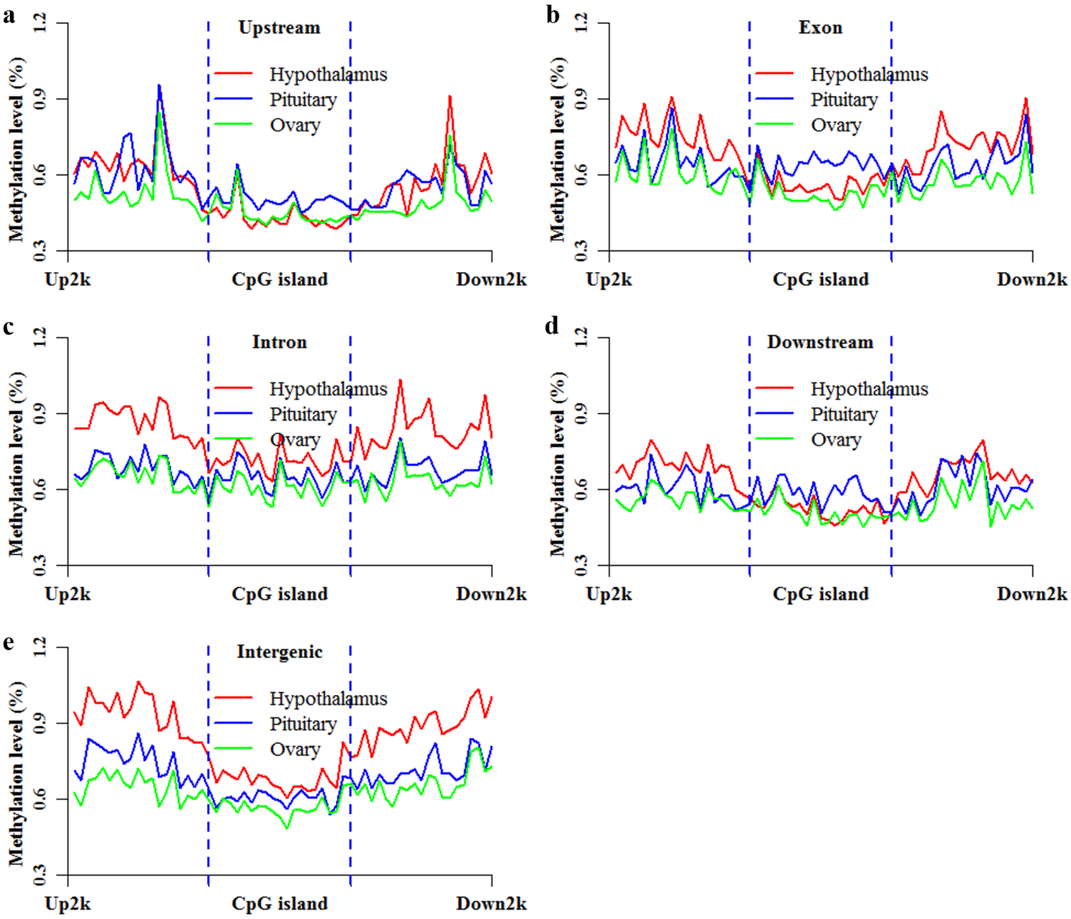

Figure S4 | Methylation patterns of CGIs located in different genic features in the CpH context. The methylation patterns of CGIs locating at upstream regions (**a**), exons (**b**), introns (**c**), downstream regions (**d**) and intergenic regions (**e**).

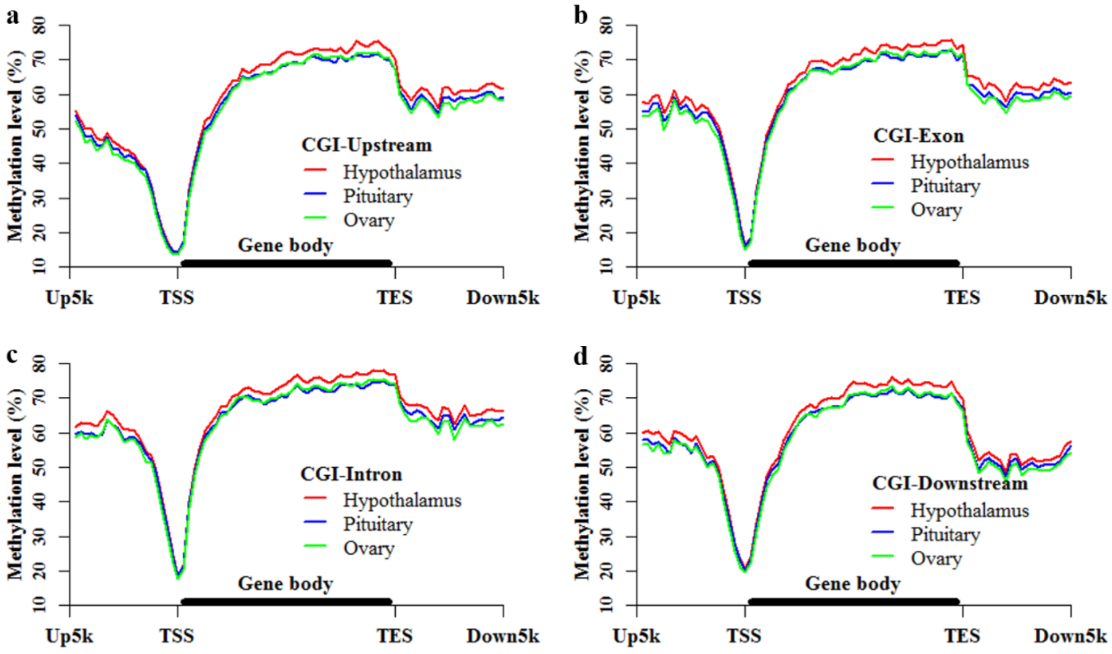

Figure S5 | Methylation patterns of genes based on different CGI locations in the CpG context. The methylation patterns of CGI-Upstream (**a**), CGI-Exon (**b**), CGI-Intron (**c**) and CGI-Downstream (**d**) genes.

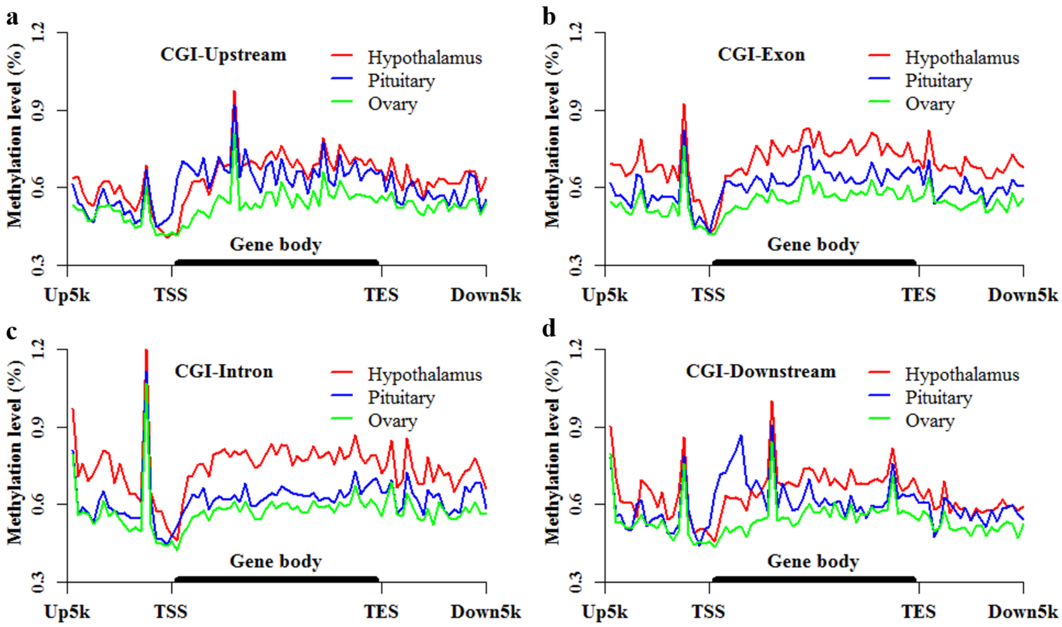

Figure S6 | Methylation patterns of genes based on different CGI locations in the CpH context. The methylation patterns of CGI-Upstream (**a**), CGI-Exon (**b**), CGI-Intron (**c**) and CGI-Downstream (**d**) genes.

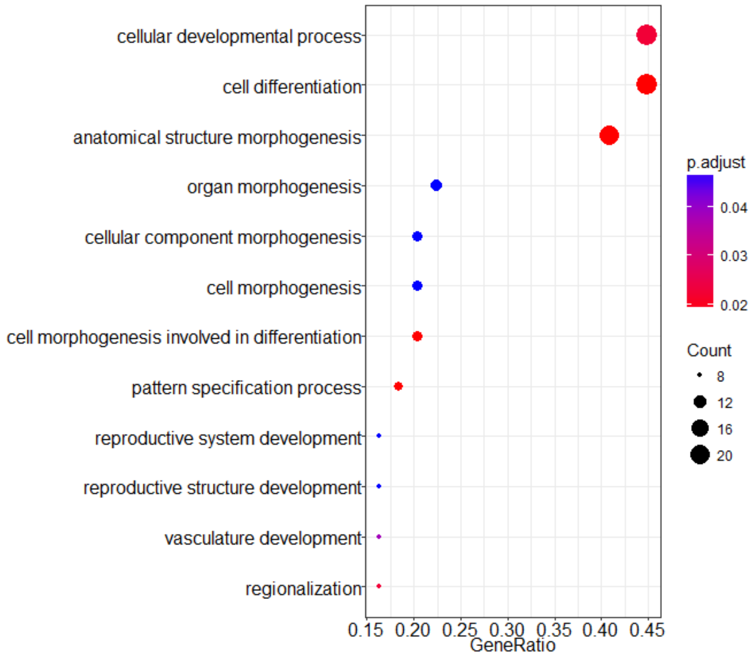

Figure S7 | Gene ontology enrichment analysis of biological processes
